# Personal

**Published:** 1889-05

**Authors:** 


### Personal.

DR. L. S. MCMURTRY, President of the Kentucky State Medical Society, has just landed from Europe, whither he has been observing professional work for the past three months. During this time, Dr. McMurtry has received much courtesy from such men as Greig Smith, Knowsley Thornton, Halliday Croom, Bantock, Sir Joseph Lister, Péan, and many others. The profession of America may be quite certain that the "ethics of medical visiting" did not suffer at the hands of the chivalrous Kentuckian, who comes from the home of McDowell, and who has done so much, not only as Secretary of the McDowell memorial but in many other ways, to perpetuate the memory of the Father ot Abdominal Surgery.

Dr. McMurtry returns in season to preside at the coming meeting of the Kentucky State Medical Society that will be held at Richmond, Ky., May 8, 9, and 10, 1889.

DR. T. M. PRUDDEN DR. HERMANN M. BIGGS and DR. H. P. LOOMIS, pathologists to the Health Department of the City of New York, have been requested by the Board of Health to formulate a brief and comprehensive statement regarding the contagiousness of tuberculosis in man, stating the evidence of the same, and recommending, in the briefest manner practicable, the simplest means of protection from its influence.

---

Dr. Prince A. Morrow, of New York, has returned from a visit to Mexico and the Sandwich Islands, whither he went to make observations concerning leprosy. He will make a report thereon to the International Congress of Dermatology and Syphilography, to be held in Paris, August 5 to 10, 1889. Dr. Morrow contributed an interesting letter to the *Journal of Cutaneous and Genito-Urinary Diseases* for April, 1889, on "Matters of Dermatological Interest in Mexico and California."

---

Professor Roswell Park, of this city, read a paper entitled, "A Study of Acute Infectious Processes in Bone," at the semi-annual meeting of the Pathological Society of Philadelphia, on the evening of April 25, 1889.

